# S46. THE VALIDITY AND SENSITIVITY OF PANSS-6 IN TREATMENT-RESISTANT SCHIZOPHRENIA

**DOI:** 10.1093/schbul/sby018.833

**Published:** 2018-04-01

**Authors:** Soren Dinesen Ostergaard, Leslie Foldager, Ole Mors, Per Bech, Christoph U Correll

**Affiliations:** 1 Aarhus University Hospital; 2 Aarhus University; 3 Psychiatric Centre North Zealand, Copenhagen University Hospital; 4 The Zucker Hillside Hospital, Northwell Health

## Abstract

**Background:**

A six-item version (PANSS-6: P1=Delusions, P2=Conceptual disorganization, P3=Hallucinations, N1=Blunted Affect, N4=Social withdrawal, N6=Lack of spontaneity/flow of conversation) of the 30-item Positive and Negative Syndrome Scale (PANSS-30) has shown promise in the measurement of symptom severity in acutely exacerbated- and chronic schizophrenia, but its validity in treatment-resistant schizophrenia remains unknown. Therefore, we tested the validity and sensitivity of PANSS-6 based on data from the clozapine phase of the Clinical Antipsychotic Trials of Intervention Effectiveness (CATIE) study.

**Methods:**

I) The scalability of PANSS-6 and PANSS-30 (i.e., whether all items provide unique information regarding syndrome severity) was tested by means of item response theory analysis ad modum Rasch; II) The correlation between PANSS-6 and PANSS-30 total scores was investigated by means of Spearman correlation analysis; III) The accuracy of PANSS-6 in identifying symptom remission was tested by comparing remission on PANSS-6 (score of ≤3 on each of the six PANSS-6 items) with remission according to the Andreasen criteria (score of ≤3 on the 8 PANSS items considered in the Andreasen criteria); and IV) The antipsychotic effect of clozapine was compared to that of olanzapine, risperidone and quetiapine using the “speed of change” on PANSS-6 and PANSS-30 (change in total score per day) as outcomes.

**Results:**

We found that I) only PANSS-6 and not PANSS-30 was scalable; II) The correlation between PANSS-6 and PANSS-30 total scores was high (Spearman coefficient: 0.85), III) PANSS-6 did accurately classify syndrome remission as defined by the Andreasen criteria, and IV) The only antipsychotic that resulted in improvement (speed of change significantly lower than 0 during the first three months of treatment) was clozapine, both when using PANSS-6 (speed of change: -0.072 points/day; 95%CI: -0.121, -0.024) and when using PANSS-30 (speed of change: -0.201 points/day; 95%CI: -0.400, -0.002) as outcome measures.

**Discussion:**

These findings suggest that PANSS-6 validly measures severity, remission and antipsychotic efficacy in treatment-resistant schizophrenia.

